# The Cellular Dysfunction of the Brain–Blood Barrier from Endothelial Cells to Astrocytes: The Pathway towards Neurotransmitter Impairment in Schizophrenia

**DOI:** 10.3390/ijms25021250

**Published:** 2024-01-19

**Authors:** Stefano Stanca, Martina Rossetti, Leona Bokulic Panichi, Paolo Bongioanni

**Affiliations:** 1 Department of Surgical, Medical, Molecular Pathology and Critical Area, University of Pisa, Via Savi 10, 56126 Pisa, Italy; 2 NeuroCare Onlus, 56100 Pisa, Italy; 3 Neuroscience Department, Azienda Ospedaliero-Universitaria Pisana, 56100 Pisa, Italy

**Keywords:** schizophrenia, blood–brain barrier, endothelial cells, permeability, astrocytes, potential therapy

## Abstract

Schizophrenia (SCZ) is an articulated psychiatric syndrome characterized by a combination of genetic, epigenetic, and environmental factors. Our intention is to present a pathogenetic model combining SCZ alterations and the main cellular actors of the blood–brain barrier (BBB): endothelial cells (ECs), pericytes, and astrocytes. The homeostasis of the BBB is preserved by the neurovascular unit which is constituted by ECs, astrocytes and microglia, neurons, and the extracellular matrix. The role of the BBB is strictly linked to its ability to preserve the biochemical integrity of brain parenchyma integrity. In SCZ, there is an increased BBB permeability, demonstrated by elevated levels of albumin and immunoglobulins in the cerebrospinal fluid, and this is the result of an intrinsic endothelial impairment. Increased BBB permeability would lead to enhanced concentrations of neurotoxic and neuroactive molecules in the brain. The pathogenetic involvement of astrocytes in SCZ reverberates its consequences on BBB, together with the impact on its permeability and selectivity represented by the EC and pericyte damage occurring in the psychotic picture. Understanding the strict interaction between ECs and astrocytes, and its consequent impact on cognition, is diriment not only for comprehension of neurotransmitter dyshomeostasis in SCZ, but also for focusing on other potential therapeutic targets.

## 1. Introduction

Schizophrenia (SCZ) is an articulated psychiatric syndrome whose faded etiologic framework is characterized by a combination of genetic, epigenetic, and environmental factors. It is notoriously explained by an intertwining of a positive and negative symptomatology, from Crow’s SCZ classification in type I (with a syndromic picture marked by a positive clinical condition) and type II (with a negative evolution of the clinical conditions) [1], and subsequent debate [2,3,4], until the last Diagnostic Statistic Manual classification.

In managing schizophrenia, the Positive and Negative Syndrome Scale (PANSS) plays a crucial role in assessing the spectrum of symptoms. This includes positive symptoms such as hallucinations, delusions, disorganized perception, cognition, and behavior, as well as negative symptoms like anhedonia, abulia, apathy, and alogia [5]. A significant aspect of schizophrenia treatment is the varying response of these symptom categories to antipsychotic medication. While positive symptoms generally show a more favorable response to therapy, negative symptoms are often more resistant to drug treatment. This differential responsiveness to antipsychotics is a key consideration in the clinical management of schizophrenia.

The use of antipsychotics, which are divided into first-generation (typical) and second-generation (atypical), is primarily aimed at correcting neurotransmitter imbalances. However, their usage is associated with a range of side effects that affect cardiovascular, metabolic, endocrinologic, and neurologic systems [6]. Neurological side effects are particularly significant, including tardive dyskinesia, extrapyramidal syndrome, and cognitive impairments associated with antipsychotic use [7].

The interplay between these adverse effects and the response of schizophrenia symptoms to treatment is complex and warrants further elaboration. Notably, the neurological and cognitive side effects of antipsychotics may aggravate negative symptoms or even mimic them, complicating the clinical picture. This exacerbation of negative symptoms by the adverse effects of antipsychotics highlights a critical challenge in treating schizophrenia while minimizing harm from side effects. It underscores the imperative to deepen our understanding of schizophrenia’s etiopathogenesis. Such insight is vital for identifying new therapeutic targets and developing more effective psychiatric medications, thereby improving treatment strategies for both positive and negative symptoms and reducing adverse effects.

Due to its complexity, several theories have been suggested to trace the biochemical complexity of this neuropsychiatric disorder. These include the dopamine hypothesis [8], which posits an imbalance in dopamine levels; the glutamate hypothesis [9], suggesting altered glutamatergic neurotransmission; and the neurodevelopmental theory [10], which implicates developmental disruptions in the etiology of the disorder.

In this review, we aim to expand beyond the established dopamine (DA) and glutamate (Glu) hypotheses associated with SCZ pathogenesis. Our focus shifts from a strictly neurocentric approach to explore a non-neuronal pathogenetic model. This model integrates structural and molecular alterations observed in the principal cellular components of the blood–brain barrier (BBB), namely endothelial cells (ECs), pericytes, and astrocytes. This exploration aims to contribute a broader understanding of SCZ etiopathogenesis. The DA-ergic theory is based on two hypotheses: the first is founded on the hyper-DA-ergic status of the subcortical structures, with particular reference to the basal ganglia; the latter, introduced to perfectionate the former, provides a combination of a mesolimbic hyper-DA-ergic status with a mesocortical hypo-DA-ergic status. Furthermore, the explanation of SCZ from the Glu perspective highlights its dysregulation in the psychotic framework with, as a consequence, a neurotoxic effect.

Building upon these theoretical foundations, this review adopts an approach that meticulously investigates peripheral factors. Specifically, it focuses on how non-neuronal elements within the brain influence neuronal activity, neurotransmitter homeostasis, and ultimately cognition. This comprehensive examination seeks to enhance our understanding of the intricate interplay between various brain components and their collective impact on cognitive processes. To evaluate the significance of these factors, it is imperative to enrich our comprehension of neuroinflammation. In the last few years, in fact, neuroinflammation has embodied a new and promising target in the neuroscientific research, from Alzheimer’s to Parkinson’s and Huntington’s diseases. Our review proposes a unitary model, combining the SCZ pathogenetic pathways from blood to neurons, through paying particular attention to the physical and biochemical interaction between ECs and astrocytes. These two cell populations are linked by being the two fundamental terms of a biomolecular complex, bridging the first-line selection of the vascular load with physical and chemical support at different levels of synaptic activity. Following this theoretical framework, the repercussions of neuroinflammation on cognition according to the glia perspective will be perused in the SCZ picture. Among the susceptibility genes associated with SCZ, there are also those codifying for cytokines such as interleukin (IL)-1B, IL-6 and IL-10 [11], with a positive correlation of IL-6 contents with the SCZ clinical picture assessed by PANSS [12], as well as between the levels of tumor necrosis factor (TNF)-α and the worsening evolution of negative symptoms [13].

Recent studies hypothesize that neuroinflammation is associated with SCZ [14]. Nevertheless, it has not been fully clarified which inflammatory pathways are involved [15]. It has been noticed that, in the first symptomatic episode of SCZ, pro-inflammatory cytokines such as IL-6 are increased, while levels of anti-inflammatory cytokines like IL-10 are decreased [16]. 

Inflammatory molecules expressed by endothelial cells have also been evaluated and it has been noticed that the vascular permeability of endothelial cells is altered together with changes in the coagulation system [17]. In addition, high levels of C-reactive protein, classically linked to inflammation, have been identified in untreated SCZ patients [18]. These elements would suggest that pro-inflammatory mechanisms would contribute to the development of a chronic inflammatory state able to damage the CNS [19]. Yet, there is no evidence that SCZ patients can take advantage of anti-inflammatory treatment [20].

This review seeks to provide clarity on these intricate relationships within the neurobiological context of SCZ [13].

By virtue of it selectively filtering blood content, the BBB is crucial in preserving brain parenchyma integrity and its neurotransmitter homeostasis from the external environment [21]. On the one hand, brain capillaries are characterized by a unique regulation of the crosstalk between the brain and the vascular system, by regulating the transit into the brain context of different molecular types, from ions, systemic metabolites, proteins, and fatty and nucleic acids [22] to drugs and exogenous agents [23,24]. On the other hand, on the neuronal side, astrocytes represent the cellular interface between the BBB and neurons. Consequently, we will try to delineate the cellular picture of the BBB, thus aiming to answer all of the questions above, facing its cellular components, ECs, pericytes, astrocytes, and microglia in the psychotic scenario: is it possible to perceive SCZ as a BBB disorder?

## 2. Endothelial Cells in Schizophrenia: When the First Line of Defense Starts to Give Up

**Figure 1:**
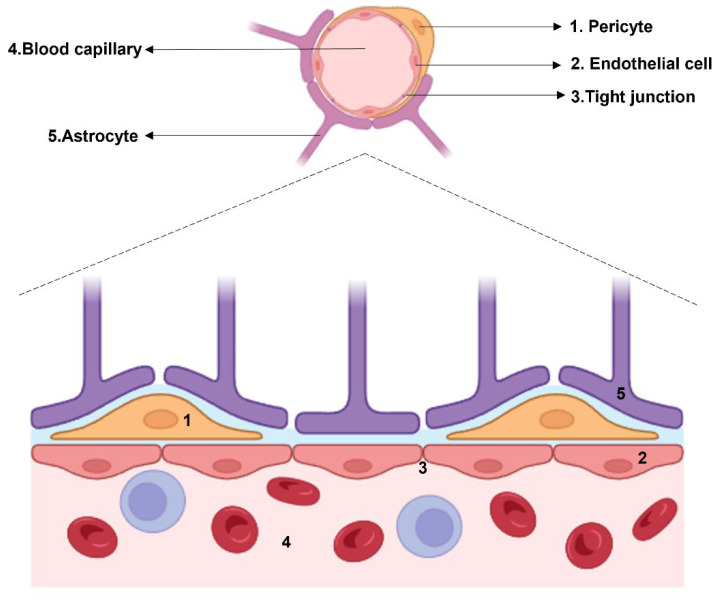
Histological structure of the BBB. The cellular components which surround the blood capillary are: pericytes, endothelial cells connected by tight junctions, and astrocytes.

The brain endothelium (Figure 1), far more than a simple barrier, is a complex, specialized structure pivotal to cognitive function. This unique aspect of the BBB [23], as first described by Ehrlich, Goldmann, and Lewandowski [24,25], is maintained by ECs in the central nervous system (CNS). These ECs, unlike those in other body areas, exhibit a higher density of tight junctions (TJs) [26,27], an absence of fenestrations, and reduced caveolar crowding, as highlighted by Ehrlich’s use of hydrophilic tracers [28] and Goldmann’s counterproofs [29]. Lewandowski’s subsequent work further clarified the importance of brain capillaries in controlling molecular flux [25,30]. In SCZ, BBB integrity becomes a focal point of concern. The BBB’s selective permeability is regulated by a complex interplay of cytokines and cellular components. Cytokines such as TNF-α and IFN-γ can impact the expression of occludin [31] and claudin in ECs [32,33,34], critical to maintaining the integrity of TJs. Astrocytes play a complementary role, as their dysfunction can lead to further BBB disruption [35]. This is particularly evident in the response of ECs and astrocytes to cytokines like IL-1β [36,37], which have been shown to affect BBB permeability and stability. The increased permeability of the BBB in SCZ, indicated by elevated albumin and IgG ratios in the CSF [38], points to a breakdown in the barrier’s selective function. This phenomenon could result from intrinsic endothelial abnormalities [39,40] or systemic influences on the BBB [41,42]. Peripheral cytokines are known to adversely affect the BBB [43], exacerbating its vulnerability in SCZ. Structural endothelial abnormalities, as noted in studies examining BBB components [44,45], further highlight the BBB’s susceptibility in this disorder. A detailed examination of the BBB in SCZ reveals an intricate relationship between ECs, immune cell activity, and cognitive processes. The heightened expression of cell adhesion molecules (CAMs) and integrins in SCZ [36,46] leads to increased lymphocyte recruitment, further implicating the immune system in the pathophysiology of SCZ. The role of ICAM-1 in the prefrontal cortex [47] and its elevated serum levels [48,49], correlated with cognitive decline, underscores the multifaceted nature of BBB dysfunction in SCZ. The broader implications of BBB alterations in SCZ extend to synaptic transmission and neuronal plasticity. Neuroinflammation, characterized by an influx of peripheral immune cells and a cascade of cytokines and chemokines [50,51,52], disrupts synaptic function and neuronal communication. This disruption is closely linked to the observed cognitive impairments in SCZ. The involvement of specific cytokines like TNF-α and IL-1β in modulating BBB permeability suggests a direct link between inflammatory processes and synaptic anomalies [53,54,55]. These findings provide a foundation for exploring therapeutic interventions targeting cytokine pathways, with the potential to mitigate both BBB dysfunction and its consequent impact on neuronal function. Moreover, the BBB’s role in SCZ is further complicated by the dynamic interactions within the neurovascular unit (NVU). The NVU, encompassing ECs, astrocytes, pericytes, and neurons, functions as a cohesive entity regulating cerebral blood flow and the neuronal microenvironment [56,57,58]. Dysregulation within the NVU, whether through cytokine imbalances or cellular dysfunction, can lead to altered neurotransmitter levels, disrupted neuronal signaling, and ultimately, the cognitive and behavioral manifestations of SCZ. The relationship between the BBB and neuroinflammation in SCZ is particularly noteworthy. Neuroinflammatory processes, including the activation of microglia and infiltration of peripheral immune cells, contribute to BBB disruption [59,60,61]. This creates a self-perpetuating cycle where BBB breakdown facilitates further neuroinflammation, exacerbating the psychiatric symptoms of SCZ. This focus is critical for understanding the potential role of cytokines in the pathogenesis of this condition. In SCZ, a disorder in which neuroinflammation and autoimmunity can play an important role in the affecting the neurotransmitter balance, cytokines have been investigated, thus bringing to light their increase in the psychosis pathogenetic framework [62,63]. TNF-α and IFN-γ levels are higher in subjects affected by SCZ as compared to controls [59,64], although these findings are not always supported [60]. In terms of cognitive performance, studies have evidenced a correlation between cytokines and cognition impairment. In this respect, in fact, PCR [61], IL-6 [65,66], TNF-α, and IL-1β are associated with a worsened cognitive performance [67]. However, given the complexity of the inflammatory picture, studies have found a correlation between TNF-α and the protection of cognitive functions [68]. Understanding these complex interactions provides crucial insights into the neurobiological underpinnings of SCZ and opens up new avenues for therapeutic intervention. In light of these considerations, future research and therapeutic strategies in SCZ must address the multifaceted nature of BBB dysfunction. This includes exploring ways to strengthen BBB integrity, modulate inflammatory responses, and restore NVU homeostasis. Potential approaches could involve targeting specific cytokine pathways, enhancing TJ protein functionality, and supporting astrocyte health to mitigate the effects of neuroinflammation on the BBB. In conclusion, the BBB’s role in SCZ is a tapestry woven from cellular interactions, cytokine dynamics, and neuroinflammatory processes, each thread contributing to the disorder’s complex pathology. A deeper understanding of these mechanisms is crucial for developing effective treatments for SCZ, moving beyond symptom management to addressing the underlying causes of this challenging condition.

## 3. Pericytes: The Cellular Interface in the Crosstalk between Endothelial Cells and Astrocytes

Pericytes, physically the closest cells to the ECs and sharing the same basal membrane, represent, together with astrocytes, an essential element in the constitution of the BBB. This is experimentally brought to light in a co-culture of a BBB syngeneic model, whose combination of pericytes and astrocytes with ECs determined the synthesis of the protein complexes involved in the formation of TJs and their consequent expression on the cellular surface [54]. ECs are linked to pericytes by N-cadherin and connexins [55]. Considering this strict association between ECs and pericytes, there is a peculiar microenvironment guaranteeing an EC–pericyte crosstalk. An increased permeability of this structure would lead to a presence in brain parenchyma of neurotoxic molecules [56], as well as neuroactive ones such as Glu itself, norepinephrine, epinephrine, and glycine (Gly) [57], leading to an increased exposure to several systemic agents involved in neuroinflammation [69]. Pericytes play a pivotal role at the neurovascular interface, providing both cerebral protection and vascular integrity. This dual function is highlighted by the occurrence of aneurysms in platelet-derived growth factor (PDGF)-deficient mice, as noted in recent studies [69]. Additionally, astrocytes constitute another critical component, significantly contributing to synaptogenesis and synaptic pruning. Their involvement underscores the complexity and importance of these cellular elements in maintaining neural and vascular health. Pericytes represent a significant interface between ECs and astrocytes in structuring NVU. They are crucial during angiogenesis, in BBB preservation, as well as in neurogenesis and neuroprotection. The NVU is essentially a network providing cellular–molecular dialogue, able to guarantee communication between the neuronal context and its environment. Pericytes are also crucial in providing extracellular vesicle (EV)-mediated intercellular communication [58,67]. The pericyte EVs are involved in the transport of nucleic acids, proteins, and fatty acids [58]. In other terms, they absolve to the transporting function, a peculiar characteristic of the BBB. Through this system of vehiculating brain-derived neurotrophic factor (BDNF), glia-derived neurotrophic factor (GDNF), and other neurotrophic factors, pericytes promote neuroprotection. In this discussion, we approach SCZ theoretically as a disorder characterized by disrupted communication between non-neuronal cells that interface the brain parenchyma with the bloodstream, thereby impacting the broader body system. A foundational aspect of this investigation involves examining the expression of specific markers, such as tetraspanins, CD8, CD9, and CD63, which are crucial for identification and understanding of these cellular interactions. This approach provides a nuanced perspective on the pathophysiology of SCZ. The above-mentioned molecules affect EVs and, consequently, neurotransmitter regulation by protein trafficking [70]. The importance of pericytes mirrors the peculiarity of EVs in the neuropsychiatric disorders. EVs, vehiculating miRNA, can act as modulator of the pathogenetic process [71,72].

## 4. Astrocytes, the Last Step before Neurons: A Cell Population between Vascularization and Cognition

Astrocytes cover an important role in structuring the synaptic network, affecting neuronal plasticity, neurotransmitter homeostasis from synaptogenesis to synaptic pruning. In the light of the *tripartite synapsis* theorization, according to which there is not only an astrocyte structural contribution to forming synapses, but also a mutual interaction between neurons and astrocytes [73], this macroglial cellular entity fulfills this role from the postnatal phase [74]. The pathogenetic involvement of astrocytes in SCZ reverberates its consequences on the BBB, together with the impact on its permeability and selectivity represented by the EC and pericyte damage occurring in the psychotic picture. In this respect, a reduction in the number of astrocytes in several brain areas from the cingulate cortex and motor cortex to the nucleus accumbens has been found [75]. Given the prominent role of astrocytes in synaptogenesis and synaptic pruning, on the one hand, their significance in the SCZ pathogenesis is clear [76] and, on the other, postulating an astrocytic disfunction in SCZ means framing SCZ as neurodevelopmental disorder in which the crucial phenomena involved in structuring the neural network are affected [77,78]. There are different levels of findings in favor of the pathogenetic role of astrocytes in the onset of SCZ, such as the existence of astrocyte-related molecules associated with the pathogenesis of SCZ [79], e.g., S100 calcium binding protein B (S100B) [80,81], whose levels are significantly higher in SCZ [82,83]; thrombospondin 1, important in synaptogenesis [84]; and serine racemase, the enzyme synthetizing D-serine, an important modulator of the ionotropic Glu *N*-methyl-D-aspartate receptor (NMDAR) [85,86]. The increased level of astrogliosis in SCZ has been marked in about 70% of SCZ subjects in postmortem investigations [75]. On a transcriptional level, an altered expression of glial fibrillary acidic protein (GFAP) mRNA has been detected in astrocytes in SCZ [87], confirmed by the reduced serum level of GFAP and S100B. Evidence of an experimental reduction in astrocytes results in a reduced spike, burst, and generally in decreased astrocyte activation [88]. In SCZ, in fact, important changes to astrocyte density and morphology have been underlined [75,89]. Therefore, it is possible to build a unitary model beyond the different findings concerning astrocytes and the GFAP levels in studies on SCZ: reactive astrogliosis is a peculiar finding in SCZ brains with enhanced levels of GFAP and S100B [75].

The reduced expression of Glu transporters in astrocytes elicits the neurotoxic effect of Glu. Astrocytes are crucial in Glu-ergic transmission: they can modulate NMDARs both positively through the D-serine pathway and negatively via kynurenic acid [75]. Due to their role in Glu metabolism, astrocytes are fundamental in the alveus of the Glu theory of SCZ. Postulated as a consequence of the psychotic-like effects of phencyclidine, this theoretical approach provides the idea that SCZ is associated substantially to a dysregulated effect of Glu, which is, as previously hinted, fundamental to the role played by astrocytes. On this matter, D-amino acid oxidase (DAO), the enzyme implicated in the catabolism of D-serine, expressed both in astrocytes and neurons [90], is overexpressed in SCZ with a consequent reduced level of D-serine and dysregulation of NMDARs [91]. D-serine is synthetized from L-serine, whose availability is the result of the activity of phosphoglycerate dehydrogenase (PHGDH), whose single-nucleotide polymorphism has been weakly associated with the onset of SCZ [92], although its impairment has been correlated to a reduced level of D-serine in the synaptic bouton [93,94]. Furthermore, due to Gly being another positive modulator of NMDARs, research has focused also on its transporter GlyT1 in astrocytes as possible target in drug therapy. This perspective has been confirmed by the antipsychotic effect GlyT-1 inhibitor sarcosine [95,96]. In confirmation of the diriment role played by astrocytes in SCZ, recent findings have also brought to light the importance of astrocytes in contributing to the equilibrium of DA [97]. Indeed, astrocytes express both D1- and D2-like receptors [98], but, more importantly, in the nucleus accumbens, their responsiveness to DA has been highlighted with a stimulation of the presynaptic ATP/adenosine receptor A1, associated with a downregulating effect on excitatory transmission [99]. The activating effect of DA on astrocytes has been evidenced also in the prefrontal cortex [100]. Finally, it is interesting to mention a study on disrupted astrocytes in schizophrenia 1 (DISC1), whose polymorphisms have been associated with SCZ [101,102]. According to this work, the elimination of such a molecule, important for neurotransmission, exclusively in astrocytes, has implications on the overexpression of D2-like receptors on the astrocytic membrane and on the downregulation of the DA-ergic activity in the basal ganglia.

## 5. Discussion

The BBB is a tight barrier affecting permeability; hence, understanding the strict cell interaction between ECs and astrocytes with the consequent impact on cognition is diriment not only to deeply comprehending neurotransmitter dyshomeostasis of SCZ, but also to focusing on potential therapeutic targets. Intimately linked to this approach is the concept of NVU representing the authentic nature of the BBB as a dynamic network from the vascular surface to neurons, linked by a bridge constituted by ECs, pericytes, and astrocytes [103]. The link between these cytotypes has been embodied by the concept of neuroinflammation investing brain parenchyma in the SCZ scenario. Starting from the ECs, we have arrived to astrocytes and their implications on neurotransmission. The objective of this work has been to build a bridge in the BBB and, as a consequence, between vascularization and neuron abnormalities in the SCZ framework.

Is there an alteration in the crosstalk between BBB ECs and astrocytes? Astrocytes affect the cerebral blood flow by impacting on the vascular caliber through the secretion of the same neurotransmitters whose dysregulation has been implied in the SCZ pathogenesis. In astrocytes, the increased intracellular Ca^2+^ is thought to be associated with the release of vasoactive substances [104]. There are, in fact, DA-ergic [92,105], Glu-ergic [106], and serotoninergic [107] astrocyte-vascular communications. The development itself of the vessels, taking place in the early development is led by the vascular endothelial growth factor (VEGF) and erythropoietin of astrocyte origin [108]. Astrocytes, beyond their traditional role in supporting neuronal function, have emerged as key players in the pathophysiology of SCZ. Recent studies have shed light on astrocytic dysfunctions, such as altered gene expression and disrupted signaling pathways, which can significantly impact synaptic homeostasis and neurotransmitter regulation. This astrocytic pathology is believed to contribute to the characteristic cognitive deficits and negative symptoms observed in SCZ. Astrocytes guarantee the normal function of the BBB through the secretion of cytokines [103]. The BBB means the creation of an environment-critical equilibrium marked by a precise extracellular level of ion and neurotransmitter levels [109]. 

Furthermore, the interaction between astrocytes and ECs is crucial to maintaining cerebral homeostasis. Dysregulation in this interaction, as seen in SCZ, can lead to impaired neurovascular coupling and cerebral blood flow, further contributing to neuronal dysfunction. There is, in fact, a closer link between ECs, pericytes, and astrocytes [109]. To peruse the alteration in this relationship between these two important poles, it is necessary to determine the molecular profile of the component of this bridge: ECs and astrocytes [23,110,111,112]. The interface represented by astrocyte end-feet and ECs embodies an important cleft to investigate, potentially pivotal in therapeutic terms [113].

## 6. Conclusions

SCZ patients exhibit cognitive impairments along with a dysfunction of the BBB. However, the impact of this alteration has not yet been fully understood. The organization of the BBB is important in selecting the transit of polar molecules, such as peptides [114], and the absent integrity of TJs in SCZ is the first aspect leading to the consideration of an increased laxity of this barrier. The BBB limits the entry of potential pathogenetic molecules; therefore, its increased permeability can provoke the deposit of neurotoxic proteins.

The current understanding of SCZ underscores the importance of an integrative approach that encompasses neuronal, astrocytic, and endothelial contributions to disease pathology. Moving beyond the traditional DA-centric view, this broader perspective highlights the complex interplay of various cell types in the brain, each contributing uniquely to the onset and progression of SCZ.

Astrocytes are the final effector of a progressive dysfunctional crosstalk inside the BBB and when they become dysfunctional they can subsequently disturb vascularization [115]. Consequently, BBB–astrocyte dysfunction may be implied in SCZ pathophysiology also, theoretically, participate in the processes leading to cognitive impairment. Even if the relationship between ECs and SCZ has not been explored in depth, it appears fundamental to further investigate it to better understand brain alterations under SCZ.

Future research should focus on unraveling the intricate mechanisms underlying astrocyte and endothelial dysfunction in SCZ. Such insights will be pivotal to developing targeted therapies that not only address the symptoms of but also the underlying cellular dysfunctions in SCZ. The potential for novel biomarkers derived from endothelial cells or astrocytes also presents an exciting avenue for early diagnosis and monitoring of SCZ.

In conclusion, the exploration of non-neuronal elements, specifically astrocytes and endothelium, in SCZ, offers promising prospects for a more effective management of this complex psychiatric disorder. Emphasizing a multi-faceted approach to understanding and treating SCZ could pave the way for breakthroughs in psychiatric care and patient outcomes.

## Data Availability

Not applicable.
